# Detection of HCV-Specific IFN-γ Responses in HCV Antibody and HCV RNA Negative Injecting Drug Users

**DOI:** 10.5812/hepatmon.14678

**Published:** 2014-01-08

**Authors:** Jacqueline K Flynn, Rachel Sacks-Davis, Peter Higgs, Campbell Aitken, Sarah Moneer, Vijay Suppiah, Lilly Tracy, Rosemary Ffrench, Scott Bowden, Heidi Drummer, Jacob George, Mandvi Bharadwaj, Margaret Hellard

**Affiliations:** 1 Centre for Biomedicine, Burnet Institute, Melbourne, Australia; 2 Department of Infectious Diseases, Monash University, Melbourne, Australia; 3 Centre for Population Health, Burnet Institute, Melbourne, Australia; 4 Department of Epidemiology and Preventive Medicine, Monash University, Melbourne, Australia; 5 National Drug Research Institute, Faculty of Health Sciences, Curtin University, Melbourne, Australia; 6 Department of Microbiology and Immunology, University of Melbourne, Parkville, Australia; 7 Storr Liver Unit, Westmead Millennium Institute, University of Sydney, Sydney, Australia; 8 School of Pharmacy, University of South Australia, Adelaide, Australia; 9 Victorian Infectious Diseases Reference Laboratory, North Melbourne, Australia; 10 Department of Immunology, Monash University, Melbourne, Australia; 11 Department of Microbiology, Monash University, Clayton, Australia

**Keywords:** Hepatitis C, Drug Users, Cohort Studies

## Abstract

**Background:**

Detectable HCV-specific cellular immune responses in HCV antibody and RNA negative people who inject drugs (PWID) raise the question of whether some are resistant to HCV infection. Immune responses from people who have been exposed to hepatitis C virus (HCV) and remain anti-HCV negative are of interest for HCV vaccine development; however, limited research addresses this area.

**Objectives:**

In a cohort of HCV antibody and RNA negative PWID, we assessed whether the presence of HCV-specific IFN-γ responses or genetic associations provide any evidence of protection from HCV infection.

**Patients and Methods:**

One hundred and ninety-eight participants were examined longitudinally for clinical, behavioral, social, environmental and genetic characteristics (IFNL3 genotype [formally IL-28B] and HLA type). Sixty-one of the 198 participants were HCV antibody and RNA negative, with 53 able to be examined longitudinally for HCV-specific IFN-γ ELISpot T cell responses.

**Results:**

Ten of the 53 HCV antibody and RNA negative participants had detectable HCV-specific IFN-γ responses at baseline (18%). The magnitude of IFN-γ responses averaged 131 +/- 96 SFC/106 PBMC and the breadth was mean 1 +/- 1 pool positive. The specificity of responses were mainly directed to E2, NS4b and NS5b. Participants with (10) and without (43) HCV-specific IFN-γ responses did not differ in behavioral, clinical or genetic characteristics (P > 0.05). There was a larger proportion sharing needles (with 70%, without 49%, P = 0.320) and a higher incidence of HCV (with 35.1 per 100 py, 95% CI 14.6, 84.4, without 16.0 per 100 py, 95% CI 7.2, 35.6, P = 0.212) in those with IFN-γ responses, although not statistically significant. Half the participants with baseline IFN-γ responses became HCV RNA positive (5/10), with one of these participants spontaneously clearing HCV. The spontaneous clearer had high magnitude and broad Th1 responses, favorable IFNL3 genotype and favorable HLA types.

**Conclusions:**

This study demonstrated the detection of HCV-specific IFN-γ responses in HCV antibody and RNA negative individuals, with a tendency for HCV-specific IFN-γ responses to be associated with HCV exposure. The potential role of HCV-specific IFN-γ responses in those who remained HCV RNA negative is of value for the development of novel HCV therapeutics.

## 1. Background

Hepatitis C virus (HCV) has infects infected an estimated 185 million people worldwide and is a significant cause of morbidity and mortality (1-3). In developed countries, HCV predominantly infects people who inject drugs (PWID) (4, 5). No vaccine for HCV is currently available with development hampered by the high degree of genetic diversity, limited animal models, and complex immunological responses to HCV (6). Recent studies have identified two groups whose immune responses are of potential interest for HCV vaccine development: spontaneous clearers and people who have been exposed to HCV but remain anti-HCV and HCV RNA negative (7, 8). 

Spontaneous clearance of acute HCV infection has been studied more extensively than those exposed to HCV who remain uninfected, and is associated with younger age, female gender, symptoms, Caucasian ethnicity and lack of co-infection with HBV and/or HIV (9, 10). The outcome of acute HCV infection is also influenced by the presence of functional CD4 + helper and CD8+ T cell responses (11, 12) (in particular the production of HCV-specific IFN-γ responses (13, 14), the innate immune response (for example CXCL10 (15) and NK activity (16, 17) and genetic factors (including HLA type (18, 19) and IFNL3 (formerly IL-28B) genotype (20). More recently combined immune (CXCL10 [IP-10]) and genetic (IFNL3) characteristics have been shown to predict spontaneous clearance (15, 21). 

Interestingly, Knapp et al. (2011) demonstrated that a polymorphism in IFNL3 (rs1297869-CC) distinguishes spontaneous clearers from exposed uninfected individuals (22). The spontaneous resolution of HCV was independently associated with IFNL3 genotype rs1297869-CC and KIR2DL3: HLA-C1, whereas exposed uninfected individuals displayed homozygosity for KIR2DL3: HLA-C1 but not for the single nucleotide polymorphism IFNL3.rs12979860. This suggests distinct immune mechanisms can influence outcomes of HCV exposure (22). 

In contrast to spontaneous clearers, there is limited research on people who have been exposed to HCV but nonetheless remain anti-HCV and HCV RNA negative. HCV-specific cellular immune responses have been observed in anti-HCV and HCV RNA negative individuals who report injecting drug use or other high-risk behaviors (23-28). Similarly, chimpanzees exposed to low doses of HCV produced HCV-specific IFN-γ ELISpot responses without viremia or seroconversion (29). Explanations proposed for this phenomenon include HCV infection followed by sero-reversion (loss of antibodies) (30, 31), HCV infection in the absence of development of antibodies (27, 32), prior HCV infection (possibly transient infection) priming effector cells generating a detectable cellular immune response without seroconversion (23) and cross-reactivity of heterologous antigens with other common pathogens (33).

Whether some people who are exposed to HCV display protective immunity is controversial. Some studies have reported a high level of HCV clearance following HCV reinfection in cohorts of PWID with reduced duration of viremia suggesting some protection against progression to chronic infection (34-37). More recently Cameron et al (2012) found the detection of HCV-specific IFN-γ responses in exposed uninfected individuals to be associated with specific risk behaviors for HCV transmission, including sharing razors and injecting anabolic steroids, and suggested that maintenance of immunity may be dependent upon regular exposure to the virus (28).

## 2. Objectives

This study longitudinally assessed three correlates of HCV clearance, HCV-specific IFN-γ responses, IFNL3 genotype and HLA type in 53 participants who were HCV antibody and HCV RNA negative. Our objective was to identify, in a longitudinal cohort of anti-HCV negative and HCV RNA negative PWID, whether the presence of IFN-γ responses or genetic associations provide any evidence of protection from HCV infection.

## 3. Patients and Methods

### 3.1. Study Participants

As described previously (38, 39), 198 PWID were recruited using modified snowball-sampled and followed between July 2005 and November 2007. Recruitment took place in three locations across Melbourne (Victoria, Australia), which were home to established street-based illicit drug market as well as a dedicated, fixed-site needle and syringe programs. Participants were recruited as part of a larger prospective cohort study on HCV primary infection, clearance and reinfection (38, 39). Participants answered behavioral questionnaires and had venous blood samples taken at baseline recruitment and at approximately three-monthly intervals (38, 39). Ethics approval was obtained from the Victorian Department of Human Services Human Research Ethics Committee (project 02/05) and the project was conducted according to the Declaration of Helsinki.

### 3.2. HCV Negative Donors

Blood samples from 15 low-risks, HCV negative donors (termed donors, with no known prior HCV exposure or risk behaviors) were obtained from the Australian Red Cross Blood Service. 

### 3.3. Serology and Virology

Blood samples were screened for anti-HCV by a third-generation enzyme immunoassay (Abbott Laboratories, Chicago, Ill). Anti-HCV positive specimens were confirmed by Murex anti-HCV version 4.0 (Murex Biotech, Kyalami, South Africa). All samples were tested for HCV RNA by COBAS AMPLICOR HCV test version 2.0 (Roche Diagnostics, Branchberg, NJ; lower limit of detection 50 IU ml-1).

IFNL3 genotyping was performed using rs8099917, rs12980275 and rs12979860 SNPs by Sequenom MassARRAY iPLEX genotyping platform as previously described (40).

### 3.4. Immunological Assays

Peripheral blood mononuclear cells (PBMCs) were separated by Ficoll-Hypaque density gradient centrifugation (Amersham–Pharmacia, Uppsala, Sweden), washed three times with phosphate-buffered saline (GIBCO BRL) and cryopreserved in 90% heat-inactivated foetal calf serum (JRH Biosciences, Kansas, USA) and 10% DMSO (Sigma-Aldrich, Castle Hill, Australia).

### 3.5. HCV Peptides

Immunological assays were performed using peptides (18aa in length overlapping by 11aa) based on the HCV genotype 1a sequence (NIH AIDS Reference and Reagent Program, Division of AIDS, NIAID, NIH: HCV 1a H77 Peptides). Peptides were grouped into pools divided by the HCV proteins (Core to NS5b, described (13, 41-43)) covering the entire HCV coding region. Peptides were titrated prior to use (optimal final concentration, 1 μg/mL (13)) and had an endotoxin level of < 0.4 EU/mL (QC-1000 LAL assay, Lonza, Melbourne, Australia (13)).

### 3.6. HCV-Specific IFN-γ ELISpot Assay

ELISpot assays were performed following manufacturer’s protocols (Mabtech, Nacka, Sweden) with the exception of the coating antibody concentration, 5 μg/mL (described (13, 41, 42)). Antigens included HCV peptide pools (1 μg/mL), positive controls (phytohemagglutinin, 5 μg/mL; Sigma-Aldrich, Sydney, Australia), Cytomegalovirus, Epstein-Barr virus, Influenza [CEF] peptides, (2 μg/mL, Mabtech), anti-CD3 antibody (2 μg/mL, Mabtech) and a negative control (RPMI with 10% FCS and 0.8% DMSO, > DMSO concentration in peptide pools). PBMCs were added to triplicate wells at 1x105 cells/well (described (13, 41-43)). Plates were incubated at 37 °C, 5% CO2 for 24 hours. Spot-forming cells (SFC) were evaluated using an automated ELISpot reader (AID version 3.2.3; Strasberg, Germany). The threshold for a positive response, ≥ 50 SFC/106 PBMC (> twice mean + 3SD, after subtraction of negative control values) were determined using 15 low-risk, seronegative blood donors with no known exposure to HCV, nor risk behaviors (13). Positive responses were always at least twice background responses.

### 3.7. Statistical Analysis

Non-parametric analysis was performed using Wilcoxon rank sum (Mann-Whitney) tests as appropriate. Kruskal Wallis and T-tests (normally distributed) were used for continuous variables and Chi-squared and Fisher’s exact tests were used for categorical variables as appropriate. Incidence rates for HCV infection were calculated using the standard person-years method with 95% confidence intervals and the midpoints of tests to estimate event dates. Exact methods were used to evaluate the differences in incidence rates. A significance level of 0.05 was used for all analyses (Stata 12.0, College Station, USA). 

## 4. Results

### 4.1. Study Participants

Between 2005 and 2007, 198 current PWID were interviewed and tested longitudinally for HCV markers. HCV exposure prevalence in this group, defined by anti-HCV antibody or HCV RNA positivity, was 69% at baseline (Appendix 1).

**Table 1.** Clinical Characteristics of Uninfected Participants With and Without HCV-Specific IFN-γ Responses at Baseline ^a^

Clinical Characteristic	Group A, IFN-γ Responses Present	Group B, IFN-γ Responses Absent	P value
**Total participants**	10	43	
**Sex ** ^**b**^			
Male	2 (20)	17 (40)	0.299
Female	8 (80)	26 (60)	
**Median age, (IQR) , y** ^**c**^	24 (18-27)	24 (21-26)	0.678
**Ethnicity ** ^**b**^			
Caucasian	8 (80)	36 (84)	1.000
Other	2 (20)	7 (16)	
**Estimated duration of injecting , y ** ^**c**^			
Median (IQR)	4 (1-9)	6 (3-9)	0.674
**Number of injections in the past month ** ^**c**^			
Median (IQR)	20 (9-26)	20 (9-41)	0.569
**Reported sharing needles ever ** ^**b**^	7 (70)	21 (49)	0.320
**Reported sharing needles in the last three months ** ^**b**^	2 (20)	10 (23)	1.000
**Main drug injected in the past three months ** ^**b**^			
Heroin	6 (60)	28 (65)	1.000
Other	4 (40)	15 (35)	
**Number received treatment for drug use** ^**d, c**^	6 (60)	28 (65)	1.000
**HLA type ** ^**e, b**^			
C_1_C_1_	1 (10)	14 (33)	0.239
C_1_C_2_	4 (40)	19 (44)	
C_2_C_2_	4 (40)	5 (9)	
***IFNL3*** ** genotype ** ^**e, b**^			
rs8099917 - TT	8 (80)	26 (60)	0.431
- GT	2 (20)	8 (19)	
- GG	0 (0)	3 (6)	
rs12980275 - AA	7 (70)	21 (49)	0.482
- GA	3 (30)	14 (32)	
- GG	0 (0)	4 (9)	
rs12979860 - CC	7 (70)	20 (47)	0.478
- CT	3 (3)	13 (30)	
- TT	0 (0)	7 (16)	
**Favourable HLA and ** ***IFNL3*** ^***b***^			
rs8099917 – TT and HLA C_1_C_1_	1 (10)	9 (21)	0.663
rs12980275 – AA and HLA C_1_C_1_	1 (10)	6 (14)	1.000
rs12979860 – CC and HLA C_1_C_1_	1 (10)	6 (14)	1.000

^a^ Number (%) reported, unless otherwise specified.

^b^ Fisher’s exact tests were used for categorical variables as appropriate. A significance level of 0.05 was used for all analyses.

^c^ Non-parametric analysis was performed using wilcoxon rank sum (Mann-Whitney) tests.

^d^ Treatment for drug use may include pharmacotherapy, detoxification, and counseling.

^e^ The HLA typing and *IFNL3* genotype does not equal 100% as some participants were untypeable. Favourable HLA type and *IFNL3* genotype are in bold.

**Figure 1.**
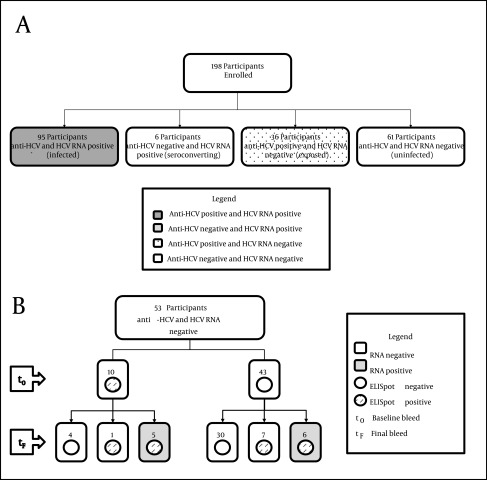
HCV RNA and Antibody Status of the 198 Study Participants A) Participants were divided by HCV RNA and HCV antibody status into four groups; both anti-HCV and HCV RNA positive termed infected (n = 95, dark grey box), anti-HCV negative and HCV RNA positive termed seroconverting (n = 3, light grey box), anti-HCV positive and HCV RNA negative termed exposed (n = 36, dotted box) and both anti-HCV and HCV RNA negative termed uninfected, (n = 61 white box). B) HCV infection and immune responses in uninfected participants. Fifty-three of the 61 uninfected participants had PBMC available at baseline (t0) and follow-up (tf) for IFN-gamma ELISpot assessment. Ten participants (Group A) had HCV-specific IFN-gamma responses at baseline, with six retaining IFN-gamma responses at follow up, and five becoming HCV RNA positive (50%). Forty-three participants did not have IFN-gamma responses at baseline (Group B) and 30 of these participants remained without IFN-gamma responses at follow up. Thirteen had detectable IFN-gamma responses at follow up, with 6 of these participants becoming HCV RNA positive (46 %).

Of the 198 participants, 95 (48%) were infected with HCV (anti-HCV and HCV RNA positive), 6 (3%) were termed seroconverting (anti-HCV negative and HCV RNA positive), 36 (18%) had evidence of past exposure to HCV (anti-HCV positive and HCV RNA negative) and 61 (31%) had no evidence of past exposure or current infection (anti-HCV and HCV RNA negative) based on standard tests at baseline (Appendix 1, Figure 1 a). 

Comparison of demographic and clinical characteristics and risk behaviors between the four groups revealed a significant difference in age (P = 0.016) ethnicity (P = 0.008), treatment for drug use (P < 0.001) and duration of injecting (P < 0.001, Appendix 1 and 2). Although not significant, there was a trend for a difference in the number of injections in the past month (P = 0.067) and sharing needles (P = 0.053), with higher proportions of participants partaking in these risk behaviors in the infected and exposed groups.

### 4.2. Uninfected Participants

The 61 uninfected participants included 53 participants (87%) with PBMCs available for longitudinal assessment of HCV-specific IFN-γ responses (baseline to follow-up, median 11 months, IQR 6-17). These 53 participants became the focus of this study (Table 1, Figure 1 b). 

Eleven uninfected participants became HCV RNA positive during the study period (Figure 1 b). This corresponded to an overall HCV incidence of 21.3 cases per 100 py (95% CI 11.8, 38.4) with the median time between enrolment and HCV RNA positive test being 12 months (IQR 8-18). 

### 4.3. HCV-Specific IFN-γ ELISpot Responses

#### 4.3.1. Baseline IFN-γ Responses

**Figure 2.**
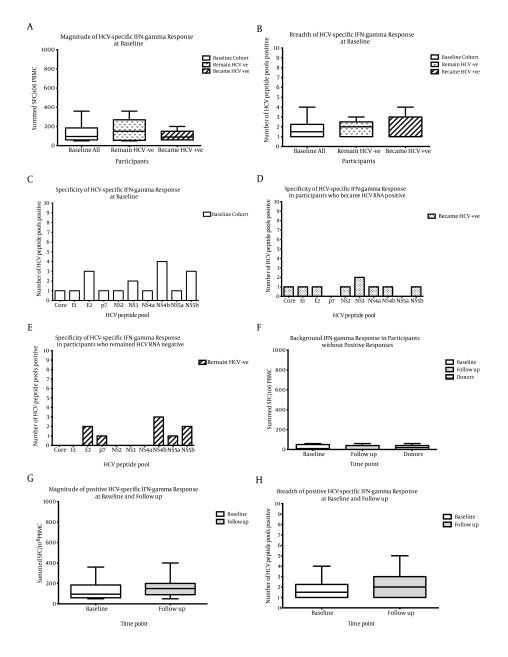
HCV-Specific IFN-Gamma Responses From HCV Uninfected Participants There was a similar A) magnitude (Mann-Whitney, P = 0.751) and B) breadth (Mann-Whitney P = 0.734) of IFN-γ responses at baseline between those who remained HCV RNA negative (dotted bars, n = 5) and those who became HCV RNA positive (hatched bars, n = 5). C) The specificity of responses were mainly directed to E2, NS4b and NS5b at baseline, with a trend for a broader specificity in D) those who became HCV RNA positive, detecting 8 different pools compared to E) those who remained HCV RNA negative. F) Background negative responses at baseline (n = 43) and follow up (n = 34) were of a similar magnitude to background responses seen in low risk control participants (termed donors, n = 15). G) The magnitude and H) breadth of positive HCV-specific IFN-gamma responses from uninfected participants at follow up were similar to baseline (n = 10 white bars, follow up (n = 8 grey bars, Mann-Whitney P > 0.200). Box and whisker plots represent the minimum to maximum values.

Ten of the 53 uninfected participants (18.8%; 95% CI 7.9-29.7) had HCV-specific IFN-γ responses at baseline, which we termed Group A (Table 1 and 2, Figure 1 b). The magnitude of IFN-γ responses averaged 131 +/- 96 SFC/106 PBMC and the breadth was mean 1 +/- 1 pool positive from a possible ten peptide pools (median magnitude 95 SFC/106 PBMC, median breadth 1 pool, Figure 2 a,b). The specificity of responses were mainly directed to E2, NS4b and NS5b (Figure 2 c).

**Table 2.** Baseline Clinical Characteristics of Group A Participants Divided by HCV RNA Status at Follow Up ^a^

Clinical Characteristic	Group A, HCV RNA Negative	Group A, HCV RNA Positive
**Total participants**	5	5
**Sex**		
Male	1 (20)	1 (20)
Female	4 (80)	4 (80)
**Median age, (IQR), y**	25 (21-31)	23 (20-27)
**Ethnicity**		
Caucasian	5 (100)	3 (60)
Other	0 (0)	2 (40)
**Estimated duration of injecting, y**		
Median (IQR)	9 (3-14)	2 (1-6)
**Number of injections in the past month**		
Median (IQR)	20 (13-35)	14 (8-27)
**Reported sharing needles ever**	3 (60)	4 (80)
**Reported sharing needles in the past three months**	0 (0)	2 (40)
**Main drug injected in the past three months**		
Heroin	4 (80)	2 (40)
Other	1 (20)	3 (60)
**Number received treatment for drug use** ^**b**^	4 (80)	2 (40)
**HLA type** ^**c**^		
C_1_C_1_	0 (0)	1 (20)
C_1_C_2_	1 (20)	3 (60)
C_2_C_2_	3 (60)	1 (20)
***IFNL3*** ** genotype** ^**c**^		
rs8099917- TT	3 (60)	5 (100)
- GT	2 (40)	0 (0)
- GG	0 (0)	0 (0)
rs12980275 - AA	3 (60)	4 (80)
- GA	2 (40)	1 (20)
- GG	0 (0)	0 (0)
rs12979860 - CC	3 (60)	4 (80)
- CT	2 (40)	1 (20)
- TT	0 (0)	0 (0)
**Favourable HLA and ** ***IFNL3***		
rs8099917 – TT and HLA C_1_C_1_	0 (0)	1 (20)
rs12980275 – AA and HLA C_1_C_1_	0 (0)	1 (20)
rs12979860 – CC and HLA C_1_C_1_	0 (0)	1 (20)

^a^Number (%) reported, unless otherwise specified.

^b^ Treatment for drug use may include pharmacotherapy, detoxification, and counseling.

^c^ The HLA typing and *IFNL3 *genotype does not equal 100% as some participants were untypeable. Favourable HLA type and *IFNL3* genotype are in bold.

Responses to each HCV peptide pool from the 43 uninfected participants without IFN-γ responses at baseline, which we termed Group B, were below the cut-off for a positive HCV-specific IFN-γ response (cut-off 50 sfc/106 PBMC, Group B mean 21 +/- 24 SFC, median 10 SFC). They were similar to background responses from 15 low-risk, HCV negative donors (termed donors, no known prior HCV exposure or risk behaviors, mean 20 +/- 20 SFC, median 20 SFC, Figure 2 f).

Demographic and clinical characteristics and risk behaviors of those with IFN-γ responses at baseline (Group A) and those without (Group B) were similar (P > 0.05, Table 1). Close examination revealed a lower median duration of injecting (Group A median 4 years, Group B median 6 years, P = 0.674) and a greater proportion sharing needles (Group A 70%, Group B 49%, P = 0.320, Table 1) in those with IFN-γ responses, although these differences were not statistically significant (Table 1). 

#### 4.3.2. Follow-up IFN-γ Responses and HCV Infection

##### 4.3.2.1. Group A

At follow-up, six of the ten Group A participants had maintained IFN-γ responses, with five of these participants being HCV RNA positive (50%; 95% CI 12-87, Table 2, Figure 1 b). Examination of the magnitude and breadth of HCV-specific IFN-γ baseline responses between participants who became HCV RNA positive (n = 5 magnitude mean 102 +/- 57 SFC and median 90 SFC, breadth mean 1 +/- 1 pool and median 1 pool) and those who remained negative (n = 5 magnitude mean 160 +/- 125 SFC and median 150 SFC P = 0.841, breadth mean 1 +/- 1 pool and median 2 pools P = 0.921) revealed they were similar (Figure 2 a, b). The specificity was broader in those who became HCV RNA positive (8 HCV pools) compared to those who remained HCV negative (5 pools, Figure 2 d, e), but it was not significant (P = 0.350). The magnitude and breadth of positive IFN-γ responses at follow-up were also similar to baseline (magnitude P = 0.443, breadth P = 0.725, Figure 2 g, h). 

The behavioural and clinical characteristics and risk behaviors of those who became HCV infected and those who remained HCV RNA negative are presented in Table 2. The estimated duration of injecting appeared to be longer for those who became HCV positive but the sample size was not sufficient to test for a statistical difference (HCV positive median 9 years, HCV negative median 2 years). 

##### 4.3.2.2. Group B

Of the 43 participants (Group B) who did not have HCV-specific IFN-γ responses at baseline, 30 did not have IFN-γ responses at follow-up (mean 19 +/- 21 SFC, median 0 SFC, similar profile to baseline background responses and those from HCV negative donors Figure 2 f). Thirteen participants had detectable IFN-γ responses, with six of these participants being HCV RNA positive (46%, Figure 1 b). The HCV-specific IFN-γ responses averaged 160 +/- 105 SFC/106 PBMC (median 150 SFC) and the breadth, mean 2 +/- 1 pools positive (median 2 pools, Figure 2 g, h). The specificity of responses was mainly directed to E2, NS4b and NS5b. The HCV positive Group B responses were not statistically different from positive Group A responses at baseline (magnitude P = 0.553, breadth P = 0.690, Figure 2 g, h). There were no significant differences between the magnitude and breadth of IFN-γ responses between HCV RNA positive and negative participants in Group B, nor between the magnitude and breath of IFN-γ responses from Group A and B at follow-up (P > 0.05). 

Behavioural and clinical characteristics and risk behaviors between those who became HCV RNA positive and those who remained negative in Group B were similar (P > 0.05, Table 3), with the exception of a higher frequency of injecting per month in participants who became HCV RNA positive (HCV positive median 50 IQR 37-61, HCV negative median 19 IQR 7-38, P = 0.016, Table 3). 

**Table 3.** Baseline Clinical Characteristics of Group B Participants Divided by HCV RNA Status at Follow Up ^a^

Clinical Characteristic	Group B, HCV RNA Negative	Group B, HCV RNA Positive	P value
**Total participants**	37	6	
**Sex ** ^**b**^			
Male	14 (37)	3 (50)	0.666
Female	23 (63)	3 (50)	
**Median age, (IQR) , y** ^**c**^	24 (21-26)	25 (22-28)	0.420
**Ethnicity ** ^**b**^			
Caucasian	32 (86)	4 (60)	0.248
Other	5 (14)	2 (40)	
**Estimated duration of injecting** ^** c**^ **, y **			
Median (IQR)	6 (3-8)	5 (1-15)	0.916
**Number of injections in the past month ** ^**c**^			
Median (IQR)	19 (7-38)	50 (37-61)	**0.016**
**Reported sharing needles ever ** ^**b**^	18 (49)	3 (50)	1.000
**Reported sharing needles in the last three months ** ^**b**^	9 (21)	1 (16)	1.000
**Main drug injected in the past three months ** ^**b**^			
Heroin	24 (65)	4 (67)	1.000
Other	13 (35)	2 (33)	
**Number received treatment for drug use** ^**d,c**^	24 (65)	4 (66)	1.000
**HLA type** ^**e,b**^			
C_1_C_1_	13 (35)	1 (16)	0.645
C_1_C_2_	17 (49)	3 (50)	
C_2_C_2_	3 (8)	1 (16)	
***IFNL3*** ** genotype** ^**e**, **b**^			
rs8099917 - TT	23 (62)	3 (50)	0.666
- GT	8 (23)	2 (33)	
- GG	3 (8)	0 (0)	
rs12980275 - AA	18 (49)	3 (50)	1.000
- GA	12 (32)	2 (33)	
- GG	4 (10)	0 (0)	
rs12979860 - CC	17 (46)	3 (50)	1.000
- CT	11 (30)	2 (33)	
- TT	6 (16)	0 (0)	
**Favourable HLA and ** ***IFNL3*** ^***b***^			
rs8099917 – TT and HLA C_1_C_1_	9 (24)	0 (0)	0.315
rs12980275 – AA and HLA C_1_C_1_	6 (16)	0 (0)	0.571
rs12979860 – CC and HLA C_1_C_1_	6 (16)	0 (0)	0.571

^a^Number (%) reported, unless otherwise specified.

^b^ Fisher’s exact tests were used for categorical variables as appropriate. Bold p values represent statistically significant results. A significance level of 0.05 was used for all analyses.

^c^ Wilcoxon rank sum (Mann-Whitney) tests

^d^ Treatment for drug use may include pharmacotherapy, detoxification, and counseling.

^e^ The HLA typing and *IFNL3 *genotype does not equal 100% as some participants were untypeable. Favourable HLA type and* IFNL3* genotype are in bold.

## 5. Discussion

Few studies have longitudinally investigated HCV-specific cellular immune responses in HCV antibody and RNA negative PWID (44). Despite ongoing injecting the majority of participants (42/53) remained HCV uninfected. Using the IFN-γ ELISpot assay as a marker of HCV exposure we found HCV-specific IFN-γ responses in a minority of participants, with half of these participants becoming HCV RNA positive within the next 12 months. 

Previous studies have used HCV-specific IFN-γ responses in HCV antibody and RNA negative participants as a marker of HCV exposure (25-28, 44), and some have postulated that the ability to mount a cellular immune response may provide some level of protection from future HCV infection (25, 26). Similar findings have been reported in chimpanzees where low doses of virus induced HCV-specific IFN-γ ELISpot responses without detectable viremia or seroconversion (29). Additional to T cell responses, the innate immune system has been suggested to play a role in prevention of HCV infection in exposed uninfected individuals, where pro-inflammatory cytokines (IL-6 and IL-8) were elevated in exposed uninfected individuals compared to spontaneous resolvers and chronic HCV participants (45). However, some of these human studies did not examine responses longitudinally and it is unknown whether the HCV status of the participants changed. 

In our study, half of the participants who had HCV-specific IFN-γ responses at baseline (Group A) were HCV infected by follow-up and half remained uninfected. It is not clear whether those who remained uninfected were due to chance or if previous HCV exposure gave some resistance to HCV infection. No key differences were identified between those who became HCV RNA positive and those who remained HCV RNA negative at baseline. However, we acknowledge a larger sample size may reveal differences in clinical and behavioural characteristics between these groups. We also cannot discount the contribution of memory T cells to the HCV-specific IFN-γ responses, it is possible a previous infection may have provided a level of resistance to persistent infection; enabling faster clearance of subsequent HCV infection (similar to what has been documented in HCV re-infection following spontaneous clearance in (34)). A longitudinal study with frequent (at least monthly) screening would enable elimination of, or provide support for, the possibility of undocumented transient infection without HCV seroconversion.

Whether HCV-specific IFN-γ responses in anti-HCV and HCV RNA negative individuals could offer protection is controversial. There has been some suggestion that a phenomenon similar to that documented in HIV could be present in HCV, in which the presence of cellular immune responses without antibody production in sex workers conferred some level of resistance to infection (46). As noted earlier, Cameron et al. (2012) suggested that maintenance of HCV immunity may be dependent upon regular exposure to the virus; in their study, significantly fewer exposed uninfected participants (with IFN-γ responses) reported a break from injecting drugs (> 6 months) compared with those who contracted HCV (28). However, in our study we did not find a difference in the frequency of injecting in those with IFN-γ responses at baseline who became HCV infected compared to those who remained HCV RNA negative. 

Cross-reactivity to other antigens has previously been raised as a possible explanation for the responses seen in anti-HCV and RNA negative participants; however, this seems unlikely to be the entire explanation in this cohort. We are confident the positive IFN-γ responses detected in this cohort are HCV-specific, as similarly to previous studies (25-27), those with positive IFN-γ responses had a diverse magnitude and breadth targeting structural and nonstructural proteins (47, 48). Nonstructural proteins are not part of the viral particle and they need to be synthesized in infected cells. This suggests participants were exposed to HCV, possibly with transient viral replication without seroconversion. Additionally, no HCV-specific IFN-γ positive responses were detected in healthy low-risk control donors, and we reduced any risk of non-specific IFN-γ production by several methods; non-specific responses were removed by subtracting any SFC found in negative control wells from the HCV peptide well count, and we had a stringent cut-off for a positive response (> twice mean + 3 SD from antigen-treated wells of healthy control donors). 

Another possible explanation for the detection of cellular immune responses in anti-HCV and RNA negative cohorts is seroreversion. However, as Zeremski et al. (2009) discussed, it is unlikely IFN-γ responses are present due to seroreversion as the young PWID in this cohort had a relatively short injection history (median seven years), whereas seroreversion usually occurs 10 years or more following spontaneous viral clearance (15, 27, 31). 

It is important to identify the potential limitations of the study. First, we did not observe any statistically significant differences in participants with or without IFN-γ responses at baseline. However, we cannot discount that a larger sample size might reveal some associations, as a higher proportion of those with IFN-γ responses at baseline (Group A) shared needles compared to those without HCV immune responses (Group B) suggesting exposure to HCV may have been higher in these participants (49, 50). Small sample size may also have limited the potential to detect associations between HCV incidence and baseline IFN-γ responses or associations between HCV RNA positivity at follow-up and other baseline characteristics. Secondly, we used genotype 1a peptides as genotype 1 is the most prevalent genotype in Australia (51, 52). Despite this and other studies observing cross-reactivity to HCV genotype 1a peptides in participants infected with other HCV genotypes (13, 42) we cannot discount our results may have underestimated the magnitude of HCV-specific IFN-γ responses. 

This study provided evidence for the detection of HCV-specific Th1 responses in HCV antibody and RNA negative PWID. Half of the participants with baseline IFN-γ responses became infected with HCV during the study, with a tendency for the incidence of HCV infection and for sharing needles to be higher in those with IFN-γ responses compared to those without. One explanation for this observation is that a positive IFN-γ response could be a surrogate marker of risky injecting and that PWID with recent exposure to HCV (inferred by IFN-γ response) are more likely to be exposed to HCV again.

In participants with IFN-γ responses who remained HCV RNA negative it remains to be clearly elucidated as to whether they had any “resistance” to HCV as there was no association with IFN-γ responses and being less likely to become infected. Future prospective studies will be important to address whether HCV-specific IFN-γ responses provide resistance to HCV infection, or decrease the duration of viremia in some individuals. Both outcomes are of considerable interest for the development of novel HCV therapeutics.

## Appendix

**Appendix 1.** Clinical Characteristics of the Entire Cohort Divided by HCV Infection and Exposure

Clinical Characteristic	Uninfected ^a^	Infected ^b^	Seroconverting^c^	Exposed^d^
**Total participants, No.**	61	95	6	36
**Sex ^e^, No. (%) **				
Male	39 (64)	61 (64)	2 (33)	23 (64)
Female	22 (36)	34 (36)	4 (67)	13 (36)
**Median age, IQR** ^******f**^ **, No. (%), y **	24 (22-27)	25 (21-27)	21 (20-23) ^g^	26 (23-35) ^g^
**Ethnicity ^ h^, No. (%) **				
Caucasian	51 (83)	62 (65) ^g^	4 (67)	32 (89)
Other	10 (17)	33 (35)	2 (33)	4 (11)
**Estimated duration of injecting ^ f^, No. (%), y **				
Median (IQR)	7 (3-10)	7 (5-11)	3 (2-4) ^g^	9 (7-15) ^i^
**Number of injections in the past month ^ f^, No. (%) **				
Median (IQR)	20 (10-40)	29 (10-70)	21 (20-45)	40 (20-61)
**Reported sharing needles ever ^ h^, No. (%) **	33 (54)	69 (73)	3 (50)	26 (74)
**Reported sharing needles in the past three months ^ h^, No. (%)**	13 (21)	31 (33)	3 (50)	7 (20)
**Main drug injected in the past three months ^ h^, No. (%)**				
Heroin	36 (60)	66 (69)	5 (100)	27 (77)
Other	24 (40)	29 (31)	0 (0)	8 (23)
**Ever received treatment for drug use ^ h, j j^, No. (%)**	40 (66)	91 (96) ^e^	3 (50)	33 (94) ^i^
**HLA type ^h, k^, No. (%)**				
C_1_C_1_	18 (33)	35 (44)	3 (50)	12 (35)
C_1_C_2_	27 (50)	34 (43)	3 (50)	19 (56)
C_2_C_2_	9 (17)	11 (14)	0 (0)	3 (9)
***IFNL3*** ** genotype ^h, k^, No. (%)**				
rs8099917 - TT	37 (67)	54 (67)	4 (80)	27 (79)
- GT	15 (27)	23 (28)	1 (20)	6 (18)
- GG	3 (5)	4 (5)	0 (0)	1 (3)
rs12980275 - AA	31 (56)	46 (57)	4 (67)	22 (63)
- GA	18 (33)	28 (35)	2 (33)	12 (34)
- GG	6 (11)	7 (9)	0 (0)	1 (3)
rs12979860 - CC	30 (55)	44 (56)	3 (60)	24 (69)
- CT	17 (31)	29 (37)	2 (40)	10 (29)
- TT	8 (15)	6 (8)	0 (0)	1 (3)
**Favourable HLA and , No. (%) ** ***IFNL3*** **, No. (%) ^ h, k^**				
rs8099917 – TT and HLA C_1_C_1_	12 (22)	23 (29)	2 (40)	10 (30)
rs12980275 – AA and HLA C_1_C_1_	9 (17)	20 (25)	3 (50)	6 (18)
>rs12979860 – CC and HLA C_1_C_1_	8 (15)	17 (22)	2 (40)	7 (21)

^a^ Uninfected participants were anti-HCV and HCV RNA negative.

^b^ Infected participants were defined as anti-HCV and HCV RNA positive.

^c^ Seroconverting participants were defined as anti-HCV negative and HCV RNA positive.

^d^ Exposed participants were defined as anti-HCV positive and HCV RNA negative.

^e^ P value for comparison with the uninfected group < 0.001.

^f^ For continuous variables, the Kruskal Wallis test was used to detect differences between all four groups. If this was statistically significant, then the Wilcoxon ranksum test was used for pairwise comparisons between groups. Please refer to Appendix 2 for pairwise comparisons.

^g^ P value for comparison with the uninfected group < 0.05.

^h^For categorical variables Fisher exact and Chi-squared tests for differences in proportion were used. First tested for differences between all four groups using Fisher exact tests and if statistically significant, pairwise comparisons between groups were made. For pairwise comparisons, a Chi-squared test was used if the expected number of participants in each cell was greater than or equal to five; otherwise Fisher’s exact tests were used.

^i^P value for comparison with the uninfected group < 0.01.

^j^ Treatment for drug use may include pharmacotherapy, detoxification, and counseling.

^k^ The HLA typing and IFNL3 genotype does not equal 100% as some participants were untypeable. Favourable HLA type and IFNL3 genotype are in bold

## Appendix

**Appendix 2.** Statistical Examination of Significantly Different Clinical Characteristics and Risk Behaviors Between the Four Groups ^a, b, c^

	Infected	Uninfected	Exposed	Seroconverting
**Age, y**				
Infected	-	0.798	0.056	**0.024**
Uninfected	-	-	**0.038**	**0.043**
Exposed	-	-	-	**0.005**
Seroconverting	-	-	-	-
**Ethnicity**				
Infected	-	**0.012**	**0.007**	1.000
Uninfected	-	-	0.474	0.291
Exposed	-	-	-	0.197
Seroconverting	-	-	-	-
**Duration of Injecting**				
Infected	-	0.064	**0.031**	**0.003**
Uninfected	-	-	**0.002**	**0.030**
Exposed	-	-	-	**0.001**
Seroconverting	-	-	-	-
**Received Treatment for Drug Use (ever)**				
Infected	-	**< 0.001**	0.660	**0.004**
Uninfected	-	-	**0.002**	0.659
Exposed	-	-	-	**0.017**
Seroconverting	-	-	-	-

^a^Pairwise comparisons P values for variables with differences between groups. Statistically significant results are in bold.

^b^For categorical variables Fisher exact and Chi-squared tests for differences in proportion were used. First tested for differences between all four groups using Fisher exact tests and if statistically significant, pairwise comparisons between groups were made. For pairwise comparisons, a Chi-squared test was used if the expected number of participants in each cell was greater than or equal to five; otherwise Fisher’s exact tests were used.

^c^For continuous variables, the Kruskal Wallis test was used to detect differences between all four groups. If this was statistically significant, then the Wilcoxon ranksum test was used for pairwise comparisons between groups.

## Appendix

**Appendix 3.** HCV Incidence in the Uninfected Cohort at Follow Up ^a^

Cohort	Cases (%)	Person-years	HCV Incidence Rate	95% CI	P value
Uninfected (n = 53)	11 (21)	51.7	21.3	11.8-38.4	
Group A (n = 10)	5 (50)	14.2	35.1	14.6-84.4	
Group B (n = 43)	6 (14)	37.5	16.0	7.2-35.6	0.212^b^

^b^Comparison between HCV incidence in Group A and Group B.

^a^Incidence rates for HCV infection were calculated using the standard person-years method with 95% confidence intervals and the midpoints of tests to estimate event dates. Exact methods were used to evaluate the differences in incidence rates.
